# Supplementary material for: Endocytosed lipids induce cell aggregation via filopodia retraction in a close relative of animals
Source: EMBO Rep. 2026 Apr 7;27(9):2274–96. doi: 10.1038/s44319-026-00760-1 (PMC13171883; doi:10.1038/s44319-026-00760-1)
Supplement: Supplementary file 2 — Movie EV1 [file 44319_2026_760_MOESM2_ESM.zip › Movie EV1/Movie EV1 legend.docx]

**Movie EV1: Aggregation of *Capsaspora* is fast.** Brightfield microscopy video of *Capsaspora* cells aggregating after induction with 5% (v/v) FBS. Continuous video taken with a MC120 HD camera. Original video was converted to a stack of frames for compression using FFmpegTool and frames from **Movie EV1** were used to generate the images in **Fig. 3A**. Scale bar is 50 µm, and time in minutes:seconds is displayed in the top left corner. Time 00:00 corresponds to the addition of FBS.
