# Supplementary material for: Endocytosed lipids induce cell aggregation via filopodia retraction in a close relative of animals
Source: EMBO Rep. 2026 Apr 7;27(9):2274–96. doi: 10.1038/s44319-026-00760-1 (PMC13171883; doi:10.1038/s44319-026-00760-1)
Supplement: Supplementary file 5 — Movie EV4 [file 44319_2026_760_MOESM5_ESM.zip › Movie EV4/Movie EV4 legend.docx]

**Movie EV4: Aggregation of *Capsaspora* coincides with uptake of fluorescent PC particles.** Confocal microscopy video of *Capsaspora* cells expressing TdTomato (red) aggregating upon addition of 100 µg/mL of fluorescent PC particles (20:1 DOPC/TopFluorPC, white). Particles localize first to filopodia and are ultimately internalized in puncta inside cells by 15 minutes. Videos generated by taking images every 4.6 seconds for 15 minutes. Scale bar is 50 µm, and time in minutes:seconds is displayed on the top left corner. Time 00:00 corresponds to the addition of fluorescent PCs.
