# Supplementary material for: Endocytosed lipids induce cell aggregation via filopodia retraction in a close relative of animals
Source: EMBO Rep. 2026 Apr 7;27(9):2274–96. doi: 10.1038/s44319-026-00760-1 (PMC13171883; doi:10.1038/s44319-026-00760-1)
Supplement: Supplementary file 6 — Movie EV5 [file 44319_2026_760_MOESM6_ESM.zip › Movie EV5/Movie EV5 legend.docx]

**Movie EV5: Uptake and aggregation induction are specific for unsaturated PC lipids.** Confocal microscopy video of *Capsaspora* cells expressing TdTomato (red) aggregating upon addition of 100 µg/mL of fluorescent PC particles (20:1 DPPC/TopFluorPC, white). Particles did not attach to the cells. They were endocytosed, and they did not induce aggregation. Video generated by taking images every 4.6 seconds for 10 minutes. Frames from this movie were used to generate the images in **Fig. 4D**. Scale bar is 20 µm, and time in minutes:seconds is displayed on the top left corner. Time 00:00 corresponds to the addition of fluorescent PCs.
