# Supplementary material for: Endocytosed lipids induce cell aggregation via filopodia retraction in a close relative of animals
Source: EMBO Rep. 2026 Apr 7;27(9):2274–96. doi: 10.1038/s44319-026-00760-1 (PMC13171883; doi:10.1038/s44319-026-00760-1)
Supplement: Supplementary file 8 — Movie EV7 [file 44319_2026_760_MOESM8_ESM.zip › Movie EV7/Movie EV7 legend.docx]

**Movies EV7: Phosphatidylcholine vesicles are trafficked along filopodia to the cell body (full field of view).** Confocal microscopy video of *Capsaspora* cells expressing the NMM-mVenus membrane marker (faint white) aggregating upon addition of 100 µg/mL of fluorescent PC particles (20:1 DOPC/TopFluorPC, bright white). Large puncta appear on the filopodia and are trafficked into the cell body in less than a minute. Video generated by taking images every 6 seconds for 6.5 minutes. White box of **Movie EV7** notes the region of the movie that was used to generate **Movie EV6** and **Fig. 5A**. Scale bar is 50 µm, and time in minutes:seconds is displayed on the top left corner. Time 00:00 corresponds to the addition of fluorescent PCs.
