# Supplementary material for: Endocytosed lipids induce cell aggregation via filopodia retraction in a close relative of animals
Source: EMBO Rep. 2026 Apr 7;27(9):2274–96. doi: 10.1038/s44319-026-00760-1 (PMC13171883; doi:10.1038/s44319-026-00760-1)
Supplement: Supplementary file 12 — Movie EV11 [file 44319_2026_760_MOESM12_ESM.zip › Movie EV11/Movie EV11 legend.docx]

**Movies EV11: Phosphatidylcholine vesicle uptake and trafficking is inhibited by Dynole 34-2.** Confocal microscopy video of *Capsaspora* cells pre-treated for 30 minutes with an endocytosis inhibitor (100 µM Dynole 34-2) and failing to aggregate upon addition of 100 µg/mL of fluorescent PC particles (20:1 POPC/TopFluorPC, bright white). No puncta appear on the filopodia or cell body, indicating failure to endocytose the vesicles. Video generated by taking images every 10 seconds for 10 minutes. Scale bar is 50 µm, and time in minutes:seconds is displayed on the top left corner. Time 00:00 corresponds to the addition of fluorescent PCs.
