# Supplementary material for: Endocytosed lipids induce cell aggregation via filopodia retraction in a close relative of animals
Source: EMBO Rep. 2026 Apr 7;27(9):2274–96. doi: 10.1038/s44319-026-00760-1 (PMC13171883; doi:10.1038/s44319-026-00760-1)
Supplement: Supplementary file 13 — Movie EV12 [file 44319_2026_760_MOESM13_ESM.zip › Movie EV12/Movie EV12 legend.docx]

**Movie EV12: Cells coalesce via retraction of interconnected filopodia, but non-connected filopodia remain outstretched (zoomed field 1).** Confocal microscopy video of *Capsaspora* cells expressing the NMM-mVenus membrane marker (white) aggregating upon addition of 100 µg/mL of (non-fluorescent) DOPC particles. Filopodia that are touching the filopodia of neighboring cells retract, pulling the cells together within 1–2 minutes. Filopodia that are not connected to other cells remain outstretched. Video generated by taking images every 6 seconds for 11 minutes. Frames from this movie were used to generate the images in **Fig. 5C**. Scale bar is 5 µm, and time in minutes:seconds is displayed on the top left corner. Time 00:00 corresponds to the addition of PCs.
