# Supplementary material for: Endocytosed lipids induce cell aggregation via filopodia retraction in a close relative of animals
Source: EMBO Rep. 2026 Apr 7;27(9):2274–96. doi: 10.1038/s44319-026-00760-1 (PMC13171883; doi:10.1038/s44319-026-00760-1)
Supplement: Supplementary file 14 — Movie EV13 [file 44319_2026_760_MOESM14_ESM.zip › Movie EV13/Movie EV13 legend.docx]

**Movie EV13: Cells coalesce via retraction of interconnected filopodia, but non-connected filopodia remain outstretched (zoomed field 2).** Confocal microscopy video of *Capsaspora* cells expressing the NMM-mVenus membrane marker (white) aggregating upon addition of 100 µg/mL of (non-fluorescenct) DOPC particles. Filopodia that are touching the filopodia of neighboring cells retract, pulling the cells together within 1–2 minutes. Filopodia that are not connected to other cells remain outstretched. Video generated by taking images every 6 seconds for 4 minutes. Frames from this movie were used to generate the images in **Fig. 5D**. Scale bar is 5 µm, and time in minutes:seconds is displayed on the top left corner. Time 00:00 corresponds to the re-addition of PCs.
