# Supplementary material for: Endocytosed lipids induce cell aggregation via filopodia retraction in a close relative of animals
Source: EMBO Rep. 2026 Apr 7;27(9):2274–96. doi: 10.1038/s44319-026-00760-1 (PMC13171883; doi:10.1038/s44319-026-00760-1)
Supplement: Supplementary file 15 — Movie EV14 [file 44319_2026_760_MOESM15_ESM.zip › Movie EV14/Movie EV14 legend.docx]

**Movie EV14: Cells coalesce via retraction of interconnected filopodia, but non-connected filopodia remain outstretched (full field of view).** Confocal microscopy video of *Capsaspora* cells expressing the NMM-mVenus membrane marker (white) aggregating upon addition of 100 µg/mL of (non-fluorescent) DOPC particles. Filopodia that are touching the filopodia of neighboring cells retract, pulling the cells together within 1–2 minutes. Filopodia that are not connected to other cells remain outstretched. Video generated by taking images every 6 seconds for 29 minutes. White boxes note the regions that were used to generate **Movies EV12–13** and **Fig. 5C–D**. Scale bar is 50 µm, and time in minutes:seconds is displayed on the top left corner. Time 00:00 corresponds to the addition of PCs (this is the start of **Movie EV12** and PCs were re-added at 20:18 which is the start of **Movie EV13**).
