# Supplementary material for: Endocytosed lipids induce cell aggregation via filopodia retraction in a close relative of animals
Source: EMBO Rep. 2026 Apr 7;27(9):2274–96. doi: 10.1038/s44319-026-00760-1 (PMC13171883; doi:10.1038/s44319-026-00760-1)
Supplement: Supplementary file 18 — Expanded View Figures [file 44319_2026_760_MOESM18_ESM.pdf]

Expanded View Figures

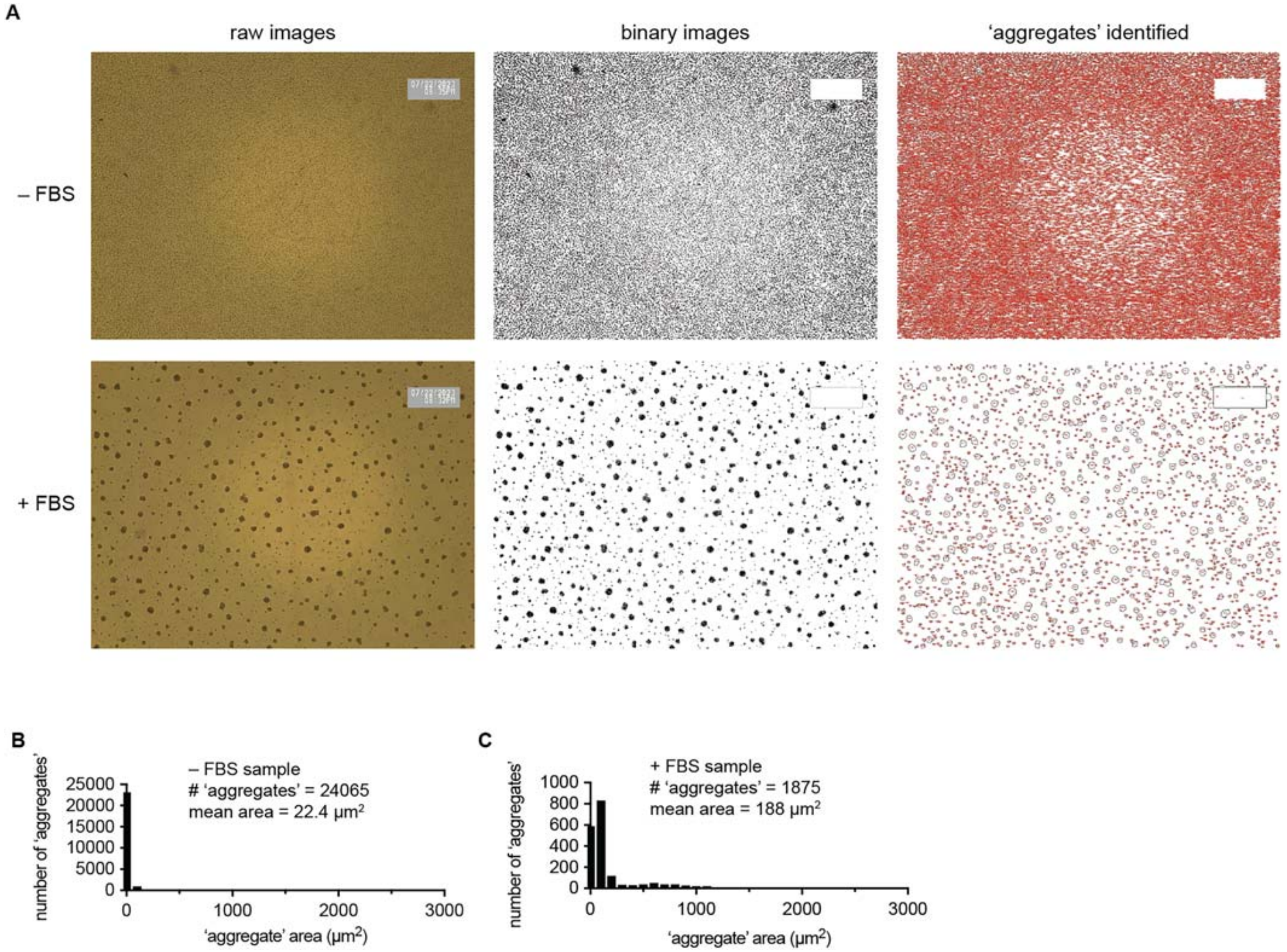

Figure EV1. Image processing illustration.

(**A**) Image analysis steps for uninduced cells (–FBS) and aggregation-induced cells (+ FBS), showing the raw images, images after binary processing, and finally the images after outlines were drawn around individual particles, which are each considered an "aggregate". (**B**) Histogram reporting the frequency distribution of "aggregates" (i.e., particles) in the −FBS sample. Nearly all "aggregates" are single cells with areas <50 μm^2. (**C**) Histogram reporting the frequency distribution of "aggregates" (i.e., particles) in the +FBS sample. In this case, most were >50 μm^2, with many near 1000 μm^2. Even though many aggregates contain hundreds of cells are present, the "mean area" for the image is only ~200 μm^2 due to the greater number of single cells and tiny aggregates. Nonetheless, "average area" distinguishes these aggregated cells from the non-aggregated condition (which had an average area ~20 μm^2).

 © The Author(s)

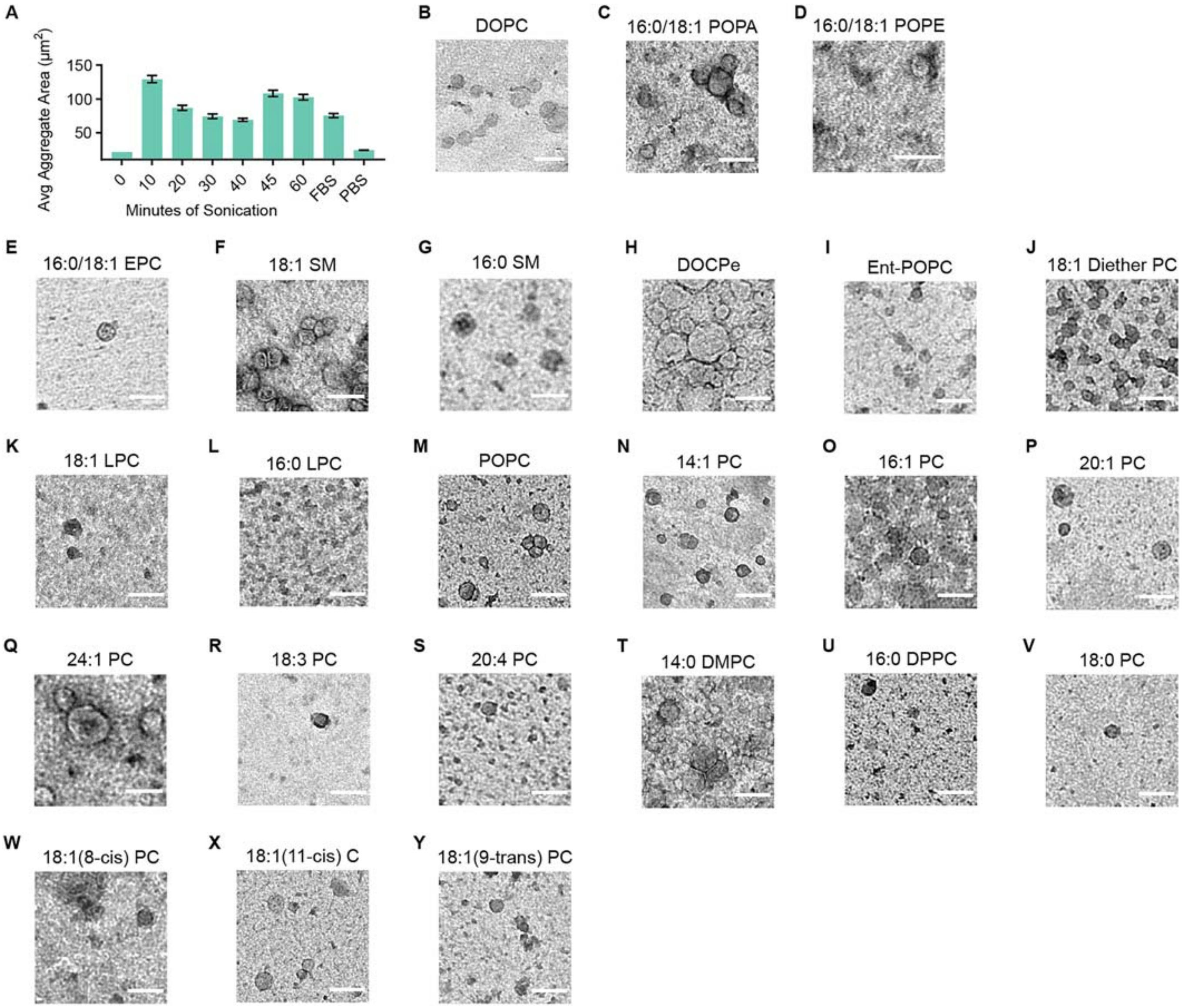

Figure EV2. Sonication and TEM images for structure–activity relationship study.

(**A**) Average aggregate area resulting from the addition of POPC prepared with varying sonication times. Preparation of PC lipids into vesicles by sonication is necessary for aggregation-induction; adding lipids without sonication failed to induce aggregation. 5% (v/v) FBS and 5% (v/v) PBS were used as positive and negative controls, respectively. Error bars represent SEM of biological triplicates ($n = 3$). For each timepoint, a single batch of sonicated vesicles was used. (**B–Y**) Transmission electron microscope (TEM) images of prepared lipid vesicles (sonicated for 10 min) stained with 1% uranyl acetate. Scale bars are 100 nm.

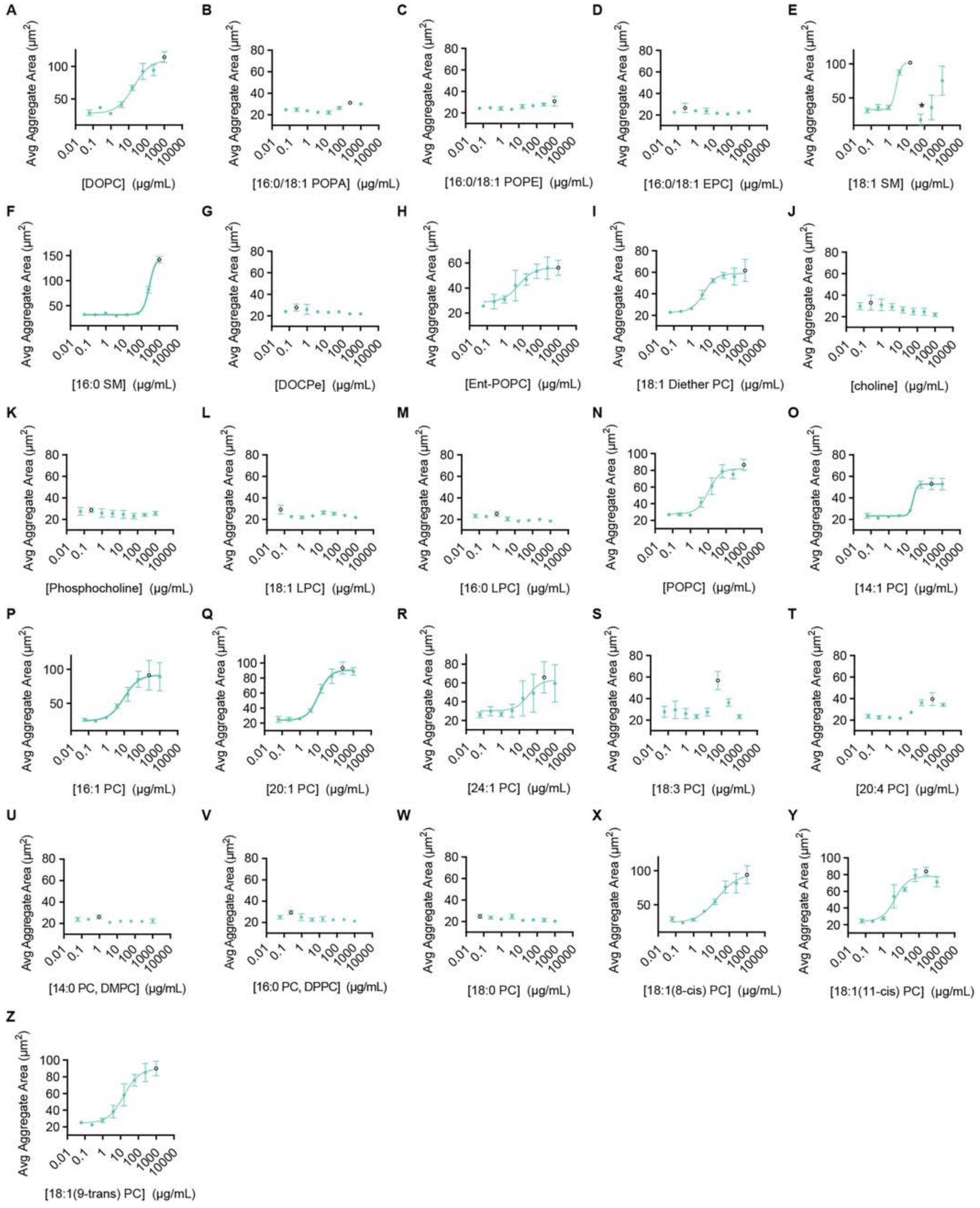
A
Avg Aggregate Area (μm²)
[DOPC] (μg/mL)
B
[16:0/18:1 POPA] (μg/mL)
C
[16:0/18:1 POPE] (μg/mL)
D
[16:0/18:1 EPC] (μg/mL)
E
[18:1 SM] (μg/mL)
F
[16:0 SM] (μg/mL)
G
[DOCPe] (μg/mL)
H
[Ent-POPC] (μg/mL)
I
[18:1 Diether PC] (μg/mL)
J
[choline] (μg/mL)
K
[Phosphocholine] (μg/mL)
L
[18:1 LPC] (μg/mL)
M
[16:0 LPC] (μg/mL)
N
[POPC] (μg/mL)
O
[14:1 PC] (μg/mL)
P
[16:1 PC] (μg/mL)
Q
[20:1 PC] (μg/mL)
R
[24:1 PC] (μg/mL)
S
[18:3 PC] (μg/mL)
T
[20:4 PC] (μg/mL)
U
[14:0 PC, DMPC] (μg/mL)
V
[16:0 PC, DPPC] (μg/mL)
W
[18:0 PC] (μg/mL)
X
[18:1(8-cis) PC] (μg/mL)
Y
[18:1(11-cis) PC] (μg/mL)
Z
[18:1(9-trans) PC] (μg/mL)

© The Author(s)

Figure EV3. Individual aggregation curves for structure activity relationship study.

(**A–Z**) Individual dose–response curves for all tested lipids (the white circle represents the largest aggregate value, which was the selected concentration reported in Fig. 1). Error bars represent SEM of biological triplicates ($n = 3$). For 18:1 SM in (**E**), high concentrations caused a single large aggregate of cells to form in some replicates. Since there was only a single aggregate, the "average" aggregate area was abnormally decreased by the individual cells in the well. This applies to concentrations greater than or equal to the point noted with an asterisk.

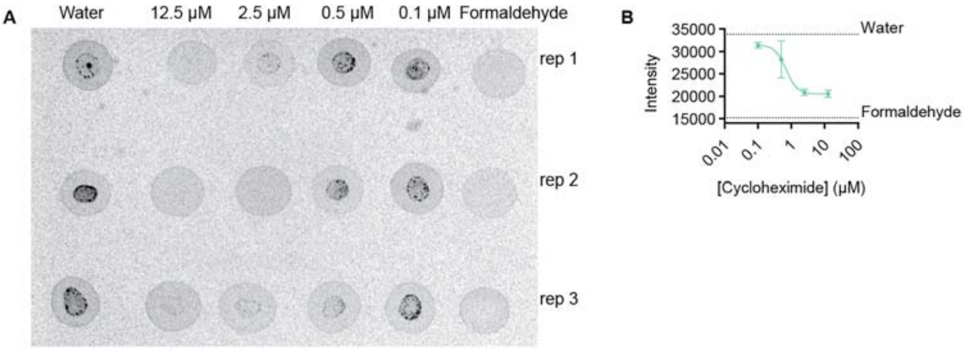

Figure EV4. Cycloheximide inhibits protein synthesis in *Capsaspora*.

(**A**) Phosphor plate image of incorporation of radioactive [^{35}S] methionine in cycloheximide-treated cells. Treated cells show no incorporation of amino acid at 12.5 µM cycloheximide, indicating no new proteins are being translated. Experiment performed in three independent replicates (n = 3). (**B**) Quantification plot of phosphor plate results. Error bars are SEM (n = 3).

 © The Author(s)

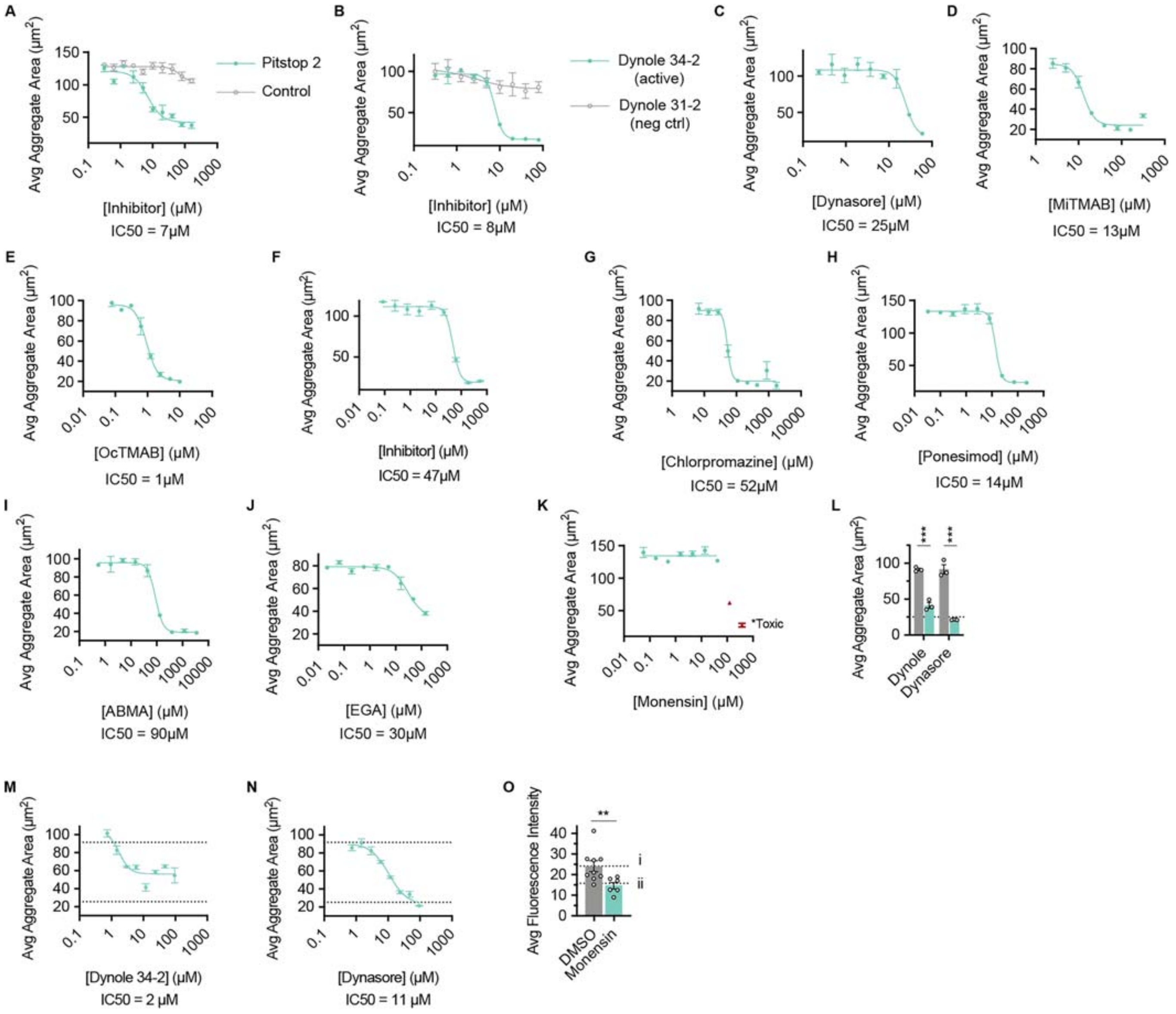

Figure EV5. Individual dose–response curves for all endocytosis inhibitors.

(**A–N**) Average aggregate areas resulting from a dilution series of various endocytosis inhibitors. (**A–K**) Aggregation was induced with 5% (v/v) FBS after 30 min of treatment with each inhibitor. (**A**) The clathrin recruitment inhibitor, Pitstop 2 inhibited aggregation while its negative control molecule did not. The IC_{50} of Pitstop in Capsaspora was found to be 7 μM. The reported IC50 for Pitstop is reported to be 12 μM in HeLa cells (von Kleist et al, 2011). (**B**) The dynamin inhibitor Dynole 34-2 (Hill et al, 2009) inhibited aggregation while its negative control molecule Dynole 31-2 did not. (**C**) The dynamin inhibitor Dynasore (Kirchhausen et al, 2008) inhibited aggregation, although not as strongly as Dynole 34-2. (**D**, **E**) The dynamin recruitment inhibitors MiTMAB and OcTMAB (Quan et al, 2007) strongly inhibited aggregation. (**F**) The actin polymerization inhibitor CK-666 (Hetrick et al, 2013) prevented aggregation. (**G**) The clathrin decoating inhibitor chlorpromazine (Vercauteren et al, 2010) inhibited aggregation. (**H**) The endosome maturation inhibitor Ponesimod (Fauzyah et al, 2021) inhibited aggregation. (**I**) The endosome maturation inhibitor ABMA (Wu et al, 2017) inhibited aggregation. (**J**) EGA even at the highest concentration of solubility still had many visible aggregates (although looser) and so was considered not active. The IC_{50} of EGA is reported to be 1 μM in A549 cells (Gillespie et al, 2013). (**K**) The lysosome pH acidification inhibitor Monensin (Misinzo et al, 2008) did not inhibit aggregation. (**L–N**) Aggregation was induced with 100 μg/mL POPC after 30 min of treatment with each inhibitor. Dashed lines indicate controls of 'no inhibitor' at the top and 'no added POPC' at the bottom. Panel (**L**) shows the most potent concentrations of the inhibitors (12 μM Dynole and 96 μM Dynasore), and panels (**M–N**) show all tested concentrations. (**O**) Average fluorescence intensity per cell of pHrodo red LDL after 30 min of treatment with the lysosome pH acidification inhibitor Monensin. Label (i) is the average intensity of cells with pHrodo staining in the absence of Monensin. Label (ii) is the average baseline fluorescence that appears as pHrodo staining in the absence of pHrodo addition. In all plots, error bars are SEM ($n = 3$). (**L**, **O**) Individual biological replicates are displayed with white circles, and each treatment was compared with its own untreated control by a *t* test. *P* values for each comparison follow: in (**L**), Dynole (0.0004), Dynasore (0.0004), and in (**O**) (0.0094).
